# *Cytomegalovirus* meningoencephalitis in a case of severe COVID-19 Pneumonia. A case report

**DOI:** 10.1016/j.idcr.2021.e01346

**Published:** 2021-11-23

**Authors:** Dore C. Ananthegowda, Mohamad Y. Khatib, Husain S. Ali, Mohamed Al wraidat, Yahia Imam

**Affiliations:** a Medical Intensive Care Unit, Hazm Mebaireek General Hospital, Hamad Medical Corporation, Doha, Qatar; b Medical Intensive Care Unit, Hamad General Hospital, Doha, Qatar; c Neuroscience Institute, Hamad Medical Corporation, Doha, Qatar

**Keywords:** CMV, Meningoencephalitis, Opportunistic infections, COVID 19, Immunosuppression, Steroids, Ganciclovir, Case report

## Abstract

The use of steroids and other immune modulatory therapies in the treatment of severe COVID-19 pneumonia predisposes patients to the reemergence of opportunistic infections. *Cytomegalovirus* (CMV) reactivation can be one of them. A 55-year-old gentleman with severe COVID-19 pneumonia and hypoxic respiratory failure who was ventilated and received steroids but no other immunomodulatory drugs; had altered sensorium and multiple episodes of seizures in the later course of his illness. Brain MRI showed leptomeningeal enhancement and encephalopathy changes, electroencephalography (EEG) was suggestive of diffuse encephalopathy and his cerebrospinal fluid (CSF) analysis revealed high Cytomegalovirus PCR DNA titers (103,614). The patient made a complete recovery after treatment with Ganciclovir. Altered sensorium in cases of COVID-19 can be multifactorial. High index of suspicion for reactivation of dormant infections is warranted. CMV meningoencephalitis is one of the differential diagnoses. We believe this is the first case reported of CMV meningoencephalitis in the setting of severe COVID-19 infection.

## Introduction

Central nervous system involvement in COVID-19 has been reported in multiple studies and reviews [1], [2], [3]. Many cases, studies and reviews specifically reported about meningoencephalitis in cases of COVID-19 [4], [5], [6]. The use of steroids and other immune modulatory therapies in the treatment of severe COVID-19 pneumonia predisposes these patients to the re-emergence of opportunistic infections [7], [8], [9]. *Cytomegalo virus* (CMV) reactivation is one of them [10], [11]. To date a literature search has not revealed any confirmed cases of CMV meningoencephalitis in the setting of COVID 19. Here we report a case of CMV meningoencephalitis in severe COVID-19 pneumonia.

## Case presentation

**Fig. 1:**
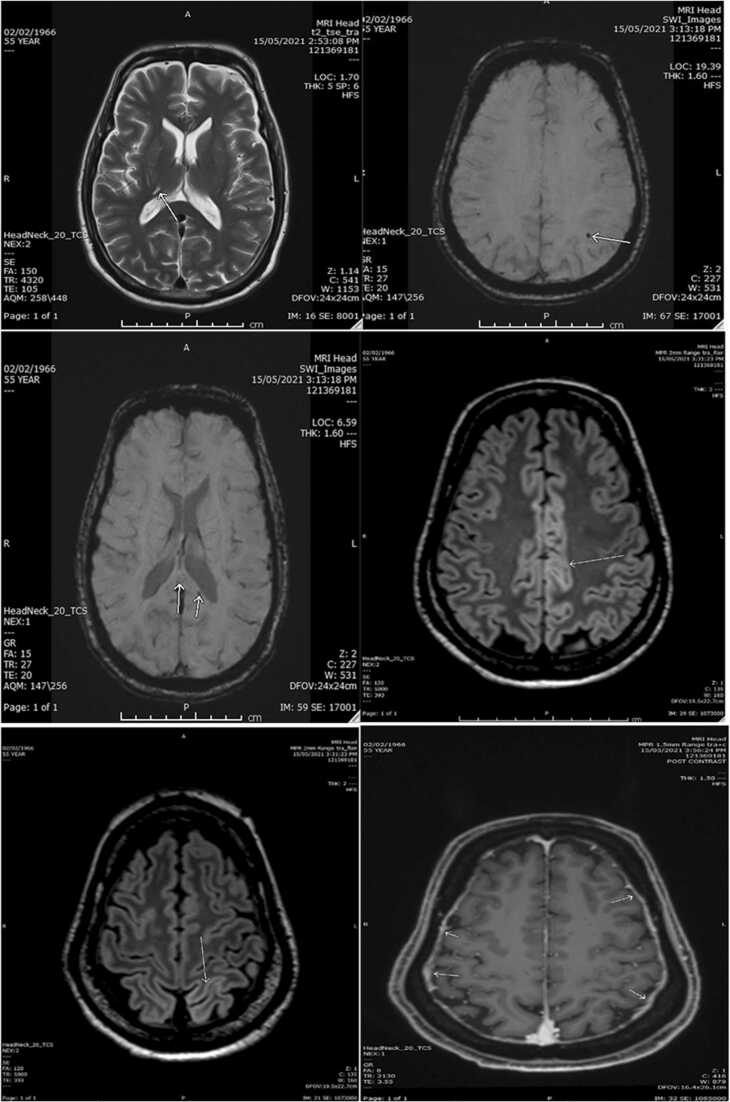
MRI Brain; Leptomeningeal Enhancement and Encephalopathy features.

A 55-year-old gentleman with a history of diabetes mellitus, systemic hypertension, hypercholesterolemia, and hyperuricemia was admitted to our Medical Intensive Care Unit (MICU)with a 10-day history of shortness of breath, fever, and productive cough. On admission he was found to have a positive COVID-19 PCR with a cycle threshold (CT) value of 21. The patient was not vaccinated against COVID-19 at the time of this hospital admission. He received treatment according to the local COVID-19 protocol. His respiratory failure was managed with oxygen supplementation via Non-re- breathing Mask, then he was shifted to high flow nasal cannula (HFNC) alternating with Continuous Positive Airway Pressure (CPAP). Pulmonary embolism was ruled out with a CT pulmonary Angiogram. As the respiratory functions deteriorated, the patient was Intubated on Day 11. On Day 20; the patient was extubated to be re-intubated again emergently for worsening respiratory failure on day 23. He was finally extubated on day 26. On day 27, the patient developed a prolonged generalized tonic-clonic seizure which lasted for about 10 min aborted with IV midazolam. A subsequent CT head was nonrevealing. Subsequently as the seizure was prolonged (status epilepticus), the patient was loaded and kept on maintenance Levetiracetam. On day 30, he developed 3 discrete episodes of seizures (each lasting approximately 2 min and responding to iv boluses of midazolam). Lacosamide was added for seizure control. The patient’s level of consciousness was not improving so he was re-intubated. His respiratory status improved, as reflected by ventilatory setting of 0.4 FIo2 and a PEEP of 5. He was kept off sedation on ventilator, but his level of consciousness did not improve. On day 34 again he developed 2 episodes of complex partial seizure that responded to IV midazolam boluses. EEG was done to rule out non convulsive status epilepticus and it showed generalized slowing suggestive of severe encephalopathy. MRI head (Fig. 1) was done and showed, scattered sporadic high cerebral mainly left sided cortical gyriform pattern with DWI bright signal with ADC map iso signal and FLAIR mild to moderate bright signal seen mainly at left high frontoparietal gyri as well as left cingulate gyrus possibly of post ictal versus early ischemic changes with no established infarction. Multiple punctate micro bleeds showing SWI dark signal are noted within the body and splenium of corpus callosum as well as left parietal subcortical regions likely of COVID-19 related micro thrombotic or hypoxic sequelae. Postcontrast, bilateral cerebral pachymeningeal dural enhancement with no nodularity is seen. Cranial and neck MRA was unremarkable.

Subsequently, lumbar puncture (LP) and cerebrospinal fluid (CSF) analysis was done on day 35 after the MRI result. CSF analysis revealed a colorless CSF with a white blood count (WBC) of 3/µl, Red blood cells (RBC) of 2425/µl, and protein was> 6 gm/L, glucose was 5.44 mmol/L. Gram stain and culture of the CSF were negative. As was the Mycobacterium tuberculosis PCR, AFB Smear and TB culture and other viral panel including EBV and Adenovirus PCRs.

CMV CSF PCR showed; 103,614 copies of the virus DNA (Test method: Altona Diagnostics Real-Time PCR assay for quantitative detection of Cytomegalovirus on ABI 7500 analyser). Subsequently, Ganciclovir treatment was started. One month later a repeat CSF analysis showed absence of CMV DNA. Other investigations including Hepatitis B, Hepatitis C, HIV and Quantiferon TB tests were negative. Connective tissue screen with antinuclear antibodies (ANA) was also negative.

Tracheostomy was done on day 48, in view of persistent low GCS and prolonged intubation.

The patient’s ICU course was complicated by *Candida Auris* and *Candida Albicans* sepsis and secondary bacterial infection with *Pseudomonas Aeruginosa* and *Klebsiella Pnemoniae* of the sputum requiring antifungal and antimicrobial treatment respectively.

Eventually the GCS improved to 15 and the patient was weaned off the ventilator and transferred to the medical ward on day 66. On day 95 he was transferred to the rehabilitation unit with persistent limb weakness.

Our patient received Dexamethasone 8 mg IV Daily D1 to D10 and Methylprednisolone 40 mg BID IV from D19 to D27.

## Discussion

We report a case of severe COVID-19 pneumonia that developed altered sensorium, new onset seizures occurring later in the disease course and was found to have encephalopathy documented electroencephalographically, abnormal CSF study and MRI brain. CMV meningoencephalitis was diagnosed based on the presence of abundant viral CMV DNA in the CSF (a key diagnostic indicator) [12] even in the absence of CSF pleocytosis that responded favourably to the treatment with IV Ganciclovir.

*Cytomegalovirus* encephalitis presenting as altered sensorium, seizures and presence of focal neurology is a rare form of CMV infection [13]. Diagnosis is usually confirmed by viral PCR. MRI findings can range from normal, to non-specific increased T2/FLAIR signal affecting cerebral white matter, to frank ventriculitis [14].

Treatment guidelines for CMV encephalitis in immunocompromised recommend combination therapy with Ganciclovir or Foscarnet [15], however monotherapy with either is reportedly adequate in non-HIV infected patients [13]. While co-infection with other pathogens is increasingly recognized in this COVID-19 pandemic [9], [16], CMV encephalitis is rare particularly in immune competent adults.

While treatment with steroids and tocilizumab [7] and hydroxychloroquine [17] may predispose patients to develop opportunistic infections due to the induced relative immunosuppression. Our patient received steroids but did not receive tocilizumab for treatment of his COVID-19 pneumonia and acute respiratory distress syndrome (ARDS) that may have predisposed the patient to CMV reactivation.

However, it is not known whether CMV reactivation per se is due to drug- related immunosuppression or due to the influence of COVID-19 influence on the immune system. The disappearance of viral DNA coupled with a good recovery maybe a clue towards a contributory influence rather than a chance discovery in our case.

## Conclusion

Our report suggests that CMV meningoencephalitis should be included in the differential diagnosis of COVID-19 patient who present with a meningoencephalitic picture by virtue of being immunologically supressed.

Early detection and treatment of CMV associated encephalitis may result in favourable outcome and would justify treatment-associated toxicity related to the administration of Ganciclovir [18].

## **Author contribution**

**Dore C. Ananthegowda:** Case collection, manuscript writing, collection of references. **Mohamad Y. Khatib:** Case collection, Manuscript review. **Husain S. Ali:** Manuscript review, collection of history. **Mohamed Al wraidat:** Case collection, ethical approval. **Yahia Imam:** Manuscript writing, collection of references and MRI Image interpretations.

## Ethical approval

Approved by Medical Research Center, HMC. Approval number: MRC-04-21-56.

## Consent

"Written informed consent was obtained from the patient for publication of this case report and accompanying images. A copy of the written consent is available for review by the Editor-in-Chief of this journal on request”.
